# The dynamic interplay of plasma membrane domains and cortical microtubules in secondary cell wall patterning

**DOI:** 10.3389/fpls.2013.00511

**Published:** 2013-12-17

**Authors:** Yoshihisa Oda, Hiroo Fukuda

**Affiliations:** ^1^Department of Biological Sciences, Graduate School of Science, The University of TokyoTokyo, Japan; ^2^ Precursory Research for Embryonic Science and Technology, Japan Science and Technology AgencySaitama, Japan

**Keywords:** secondary cell wall, ROP GTPase, MIDD1, cortical microtubule, xylem

## Abstract

Patterning of the cellulosic cell wall underlies the shape and function of plant cells. The cortical microtubule array plays a central role in the regulation of cell wall patterns. However, the regulatory mechanisms by which secondary cell wall patterns are established through cortical microtubules remain to be fully determined. Our recent study in xylem vessel cells revealed that a mutual inhibitory interaction between cortical microtubules and distinct plasma membrane domains leads to distinctive patterning in secondary cell walls. Our research revealed that the recycling of active and inactive ROP proteins by a specific GAP and GEF pair establishes distinct *de novo* plasma membrane domains. Active ROP recruits a plant-specific microtubule-associated protein, MIDD1, which mediates the mutual interaction between cortical microtubules and plasma membrane domains. In this mini review, we summarize recent research regarding secondary wall patterning, with a focus on the emerging interplay between plasma membrane domains and cortical microtubules through MIDD1 and ROP.

## INTRODUCTION

The cellulosic cell wall plays a central role in shaping cells and determining cell function in plants. For example, physically rigid cellulose microfibrils restrict the direction of cell expansion and lead to the distinct shape of plant cells. Cortical microtubules play a central role in regulating the development of cell wall structures by controlling how the cellulose synthase complex is targeted to the plasma membrane (Paredez et al., 2006; Crowell et al., 2009; Gutierrez et al., 2009). Although cortical microtubules are closely and tightly anchored to the plasma membrane, they exhibit dynamic behaviors, such as growth, shrinkage, and branching (Shaw et al., 2003; Murata et al., 2005; Chan et al., 2009; Nakamura et al., 2010) and exhibit bundling (Dixit and Cyr, 2004), which leads to the self-organization of parallel microtubule arrays (Wasteneys and Ambrose, 2009). Several microtubule-associated proteins (MAPs) have been discovered that regulate the global dynamics of cortical microtubules in the cell. However, the molecular mechanisms underlying local regulation of cortical microtubule dynamics, for formation of structures such as patterned secondary cell walls in xylem vessels, are not well understood.

Xylem vessels are water-conductive tubes composed of dead cells called tracheary elements. Differentiating xylem cells deposit rigid water-impermeable secondary cell walls to avoid collapse of the vessel due to the negative pressure exerted as a consequence of water transport. In xylem vessels, secondary cell walls are deposited in various patterns, such as annular, spiral, reticulate, and pitted. Usually, protoxylems develop annular and spiral secondary walls while metaxylems exhibit reticulate and pitted formations. Perforations in the end walls of xylem cells and secondary wall pits in the side walls contribute to effective longitudinal and lateral water transport, respectively. Thus, the patterning of secondary cell walls is tightly coupled to function in xylem vessels (Esau, 1977).

The involvement of cortical microtubules in the regulation of secondary cell wall deposition during xylem vessel differentiation has been revealed through a number of studies. Dense cortical microtubule bundles are found beneath secondary wall thickenings and, if these cortical microtubules are pharmacologically depolymerized, the secondary cell wall patterns become severely disorganized (Hepler, 1981; Gunning and Hardham, 1982). Direct visualization of the cellulose synthase complex confirmed that patterned cortical microtubules regulate the localization of the cellulose synthase complex in xylem vessels (Wightman and Turner, 2008). Studies using isolated zinnia mesophyll cells revealed that a dramatic rearrangement of cortical microtubules leads to distinct patterns of secondary cell walls during tracheary element differentiation (Falconer and Seagull, 1985, 1986, 1988; Kobayashi et al., 1987, 1988; Fukuda and Kobayashi, 1989). Recent live cell imaging of differentiating xylem cells using *Arabidopsis* cell cultures further revealed that bundling and disassembly of cortical microtubules gives rise to distinct patterns of secondary cell walls (Oda et al., 2005, 2010; Oda and Hasezawa, 2006; Pesquet et al., 2010). These studies also revealed that several MAPs regulate the dynamic behavior of cortical microtubules during xylem differentiation (Oda et al., 2010; Pesquet et al., 2010; Oda and Fukuda, 2012b, 2013b). In addition, a recent study revealed that ROP GTPases generate plasma membrane domains that play critical roles in the spatial regulation of cortical microtubule dynamics, and that mutual inhibitory interactions between the plasma membrane domains and cortical microtubules establishes distinct patterns of secondary cell walls (Oda and Fukuda, 2012a). In this review, we focus on the emerging role of the dynamic interplay between plasma membrane domains and cortical microtubules in secondary cell wall patterning.

## MICROTUBULE-DEPOLYMERIZING PLASMA MEMBRANE DOMAIN LEADS SECONDARY WALL PIT

We previously established an *Arabidopsis* cultured cell line for *in vitro* xylem differentiation (Oda et al., 2010). In this system, VND6, a master transcription factor that prompts metaxylem vessel differentiation (Kubo et al., 2005), was introduced under the control of an estradiol inducible promoter (Zuo et al., 2000), allowing synchronous induction of metaxylem vessel differentiation at high frequency. Using this system, we found that there are several cellular regions in which cortical microtubules are unstable. Cortical microtubules are eventually depleted in these regions, resulting in the formation of secondary wall pits. This suggests that local depolymerization of cortical microtubules is the key event for formation of secondary wall pits (Oda et al., 2010). Cortical microtubules are closely anchored to the plasma membrane (Hardham and Gunning, 1978; Seagull and Heath, 1980; Lancelle et al., 1986; Giddings and Staehelin, 1988), and specific regulators of microtubule dynamics are thus expected to be locally present at the plasma membrane, mediating the formation of microtubule-depolymerizing plasma membrane domains.

## MIDD1 IS LOCALIZED TO MICROTUBULE-DEPLETING PLASMA MEMBRANE DOMAINS

Microtubule-associated protein MIDD1 (microtubule depletion domain 1) is preferentially associated with depolymerizing cortical microtubules in the future pit region of secondary walls in xylem cells. Knockdown of MIDD1 inhibits the local disassembly of cortical microtubules, resulting in the loss of secondary wall pits. Conversely, overexpression of MIDD1 in non-xylem cells reduces cortical microtubule density. MIDD1 is composed of two coiled-coil domains: the N-terminal domain, which binds directly to microtubules, and the C-terminal domain, which is associated with specific plasma membrane domains. MIDD1 is thus thought to promote depolymerization of cortical microtubules in the plasma membrane domains, leading to the formation of secondary wall pits (Oda et al., 2010).

## MIDD1 RECRUITS AtKinesin-13A TO LOCALLY DEPOLYMERIZE CORTICAL MICROTUBULES

**FIGURE 1:**
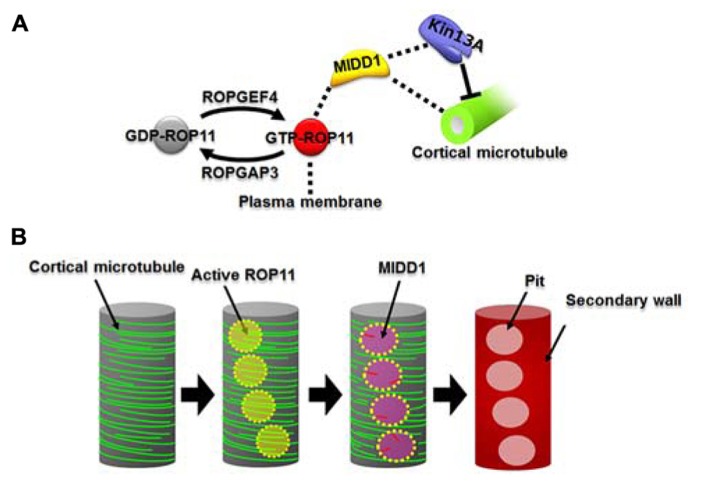
**The ROP11-MIDD1 pathway produces the microtubule-depolymerizing domain.** **(A)** MIDD1 links plasma membrane, cortical microtubules, and AtKinesin-13A. ROP11 is locally activated by ROPGEF4 and ROPGAP3. The activated ROP11 (GTP-ROP11) recruits MIDD1 to the plasma membrane, which in turn binds cortical microtubules. MIDD1 subsequently recruits AtKinsein-13A (Kin13A), which depolymerizes the cortical microtubule. **(B)** Locally activated ROP11 recruits MIDD1 to the plasma membrane domains. MIDD1 subsequently induces microtubule disassembly by recruiting AtKinesin-13A, resulting in the formation of secondary wall pits.

Microtubule depletion domain 1 does not exhibit *in vitro *microtubule depolymerization activity. MIDD1 is therefore likely to interact with other proteins to induce microtubule disassembly. Recently, AtKinsein-13A was found to interact with MIDD1 in yeast (Mucha et al., 2010). AtKinesin-13A belongs to the kinesin-13 family (Lu et al., 2005), whose animal members have microtubule depolymerization activity (Desai et al., 1999). As expected, AtKinesin-13A exhibits microtubule depolymerization activity *in vitro* (Oda and Fukuda, 2013a). Knockdown of *AtKinesin-13A* results in smaller secondary wall pits, while overexpression of *AtKinesin-13A* enlarges secondary wall pits. AtKinesin-13A co-localizes and interacts with MIDD1 in secondary wall pits and promotes depolymerization of cortical microtubules. In the absence of MIDD1, however, AtKinsein-13A neither localizes to nor affects cortical microtubules (Oda and Fukuda, 2013a). Therefore, it is likely that MIDD1 is a scaffold protein that links the plasma membrane domains to AtKinesin-13A and therefore facilitates specific localized cortical microtubule depolymerization (**Figure 1A**).

## ROP GTPase REGULATES CORTICAL MICROTUBULE DYNAMICS THROUGH THE MIDD1-AtKinesin-13A COMPLEX

Microtubule depletion domain 1 belongs to the RIP/ICR family, members of which interact with the active forms of ROP GTPases via their conserved C-terminal motif (Lavy et al., 2007; Li et al., 2008). ROP11 distributes broadly at the plasma membrane and, together with MIDD1, accumulates specifically on cortical microtubules in the secondary wall pits (Oda and Fukuda, 2012a). Further cellular analysis revealed that the active form of ROP11 recruits MIDD1 to the plasma membrane. Introduction of a constitutively activated mutant ROP11 strongly inhibits formation of secondary wall pits in metaxylem vessels by affecting pit-specific localization of AtKinesein-13A (Oda and Fukuda, 2012a, 2013a). These results suggest that locally activated ROP11 is essential for formation of secondary wall pits through the recruitment of the MIDD1-AtKinesin-13A complex (**Figure 1B**).

## ROP GTPases GENERATE MICROTUBULE-DEPOLYMERIZING PLASMA MEMBRANE DOMAINS

ROP11 is specifically active at the secondary wall pits, posing the question of how regulation of this local activation is achieved. ROP GTPases are activated by plant-specific guanine nucleotide exchange factors (ROPGEFs) and are inactivated by ROP GTPase-activating proteins (ROPGAPs; Wu et al., 2000; Berken et al., 2005; Gu et al., 2006). ROPGAP3 and ROPGEF4 mediate the local activation and inactivation, respectively, of ROP11 in differentiating xylem cells (Oda and Fukuda, 2012a). Both ROPGEF4 and ROPGAP3 localize to plasma membrane domains in secondary wall pits. Interestingly, ROPGEF4 is concentrated at the center of the domains, while ROPGAP3 localizes much more broadly in the secondary wall pits. Knockout or knockdown of *ROPGEF4* causes reduced density of secondary wall pits in metaxylem vessels, suggesting that ROPGEF4 promotes formation of secondary wall pits through local activation of ROP11 (**Figure 1A**; Oda and Fukuda, 2012a). Co-expression of ROP11, ROPGEF4, and ROPGAP3 in non-xylem cells causes evenly spaced patches of ROPGEF4 at the plasma membrane, which in turn activates ROP11 around the patches. This locally activated ROP11 recruits MIDD1 and finally causes local disassembly of cortical microtubules (Oda and Fukuda, 2012a).

The next question that arises is how such spontaneously activated ROP11 domains might be formed. Loss of either ROPGEF4 or ROPGAP3 abolishes the activated ROP11 domains. Furthermore, constitutively active or negative mutants of ROP11 cannot mediate this event (Oda and Fukuda, 2012a). Therefore, GTP-GDP cycling of ROP11 by ROPGEF4 and ROPGAP3 is essential for the self-organization of the activated ROP11 domains. Neither microtubules nor actin microfilaments are required for domain formation. A possible mechanism to explain this spontaneous formation of active ROP11 patches is positive feedback from active ROP11 via ROPGEF4 as some receptor-like kinases are known to interact with ROPGEFs (Zhang and McCormick, 2007; Duan et al., 2010; Chang et al., 2012; Akamatsu et al., 2013). For example, AtPRK2, a receptor-like kinase, which functions in polarized growth of pollen tubes, interacts with both ROPGEF1 and ROP GTPases, and phosphorylates ROPGEF1 to activate ROP GTPase (Chang et al., 2012). Similarly, an unknown receptor-like kinase(s) might interact with ROPGEF4 and ROP11 to initiate positive feedback and consequently form activated ROP11 patches. A negative feedback mechanism between ROPGAP3 and ROPGEF4 via ROP11 may also contribute to the self-organization of activated ROP11 patches.

## CORTICAL MICROTUBULES ACT AS A FENCE INHIBITING THE MOVEMENT OF THE ROP-MIDD1 COMPLEX

Another important finding with respect to the formation of localized secondary wall pits is that cortical microtubules restrict the localization of active ROP11. Treatment with taxol, which stabilizes microtubules, elongates active ROP11 domains, suggesting that cortical microtubules can affect the localization of active ROP11 (Oda and Fukuda, 2012a). Further reconstruction experiments in non-xylem cells revealed that active ROP11 domains are enclosed by cortical microtubules to form polygonal structures. However, disruption of cortical microtubules by chemical treatment with oryzalin or co-expression of AtKinesin-13A resulted in round active ROP11 domains. These results strongly suggest that cortical microtubules act as a fence preventing outward diffusion of active ROP11 (Oda and Fukuda, 2012a).

**FIGURE 2:**
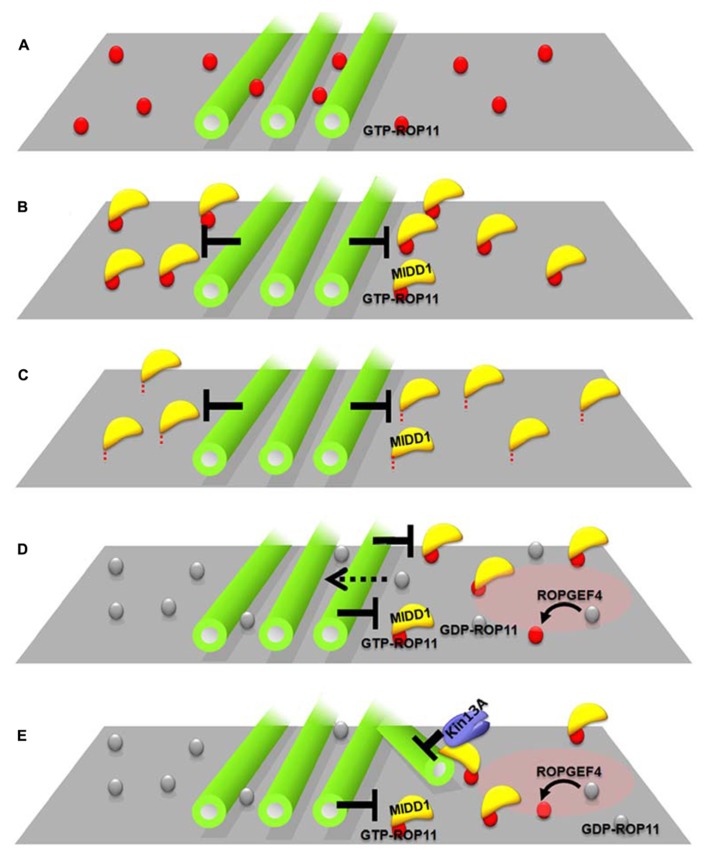
**Interplay between cortical microtubules and plasma membrane domains via the ROP11-MIDD1 complex.** **(A–C)** MIDD1 mediates elimination of active ROP11 by cortical microtubules. **(A)** Cortical microtubules do not affect the localization of active (GTP-) ROP11. **(B)** Cortical microtubules eliminate active ROP11-truncated MIDD1 complex from the plasma membrane (truncated mutant lacking microtubule-binding domain). **(C)** Cortical microtubules eliminate the membrane–anchored truncated MIDD1. **(D,E)** Interplay between cortical microtubules and plasma membrane domains in xylem cells. **(D)** Cortical microtubules restrict the plasma membrane domain of active ROP11 generated by ROPGEF4. Inactive ROP11 is not affected by cortical microtubules, because there is no interaction with MIDD1. **(E)** Active ROP11-MIDD1-AtKinesin-13A complex associates with the microtubule growing into the domain and induces depolymerization.

How does MIDD1 contribute to this phenomenon? In non-xylem cells, cortical microtubules restricted active ROP11-MIDD1 domains when truncated MIDD1, which lacks its microtubule-binding domain, was introduced with ROP11, ROPGEF4, and ROPGAP3 (**Figure 2B**). In the absence of MIDD1, however, cortical microtubules did not affect the localization of ROP11 (**Figure 2A**). In the absence of ROP11, truncated MIDD1 was distributed broadly in the cytoplasm and its distribution was unaffected by cortical microtubules (Oda and Fukuda, 2012a). By contrast, cortical microtubules could eliminate truncated MIDD1 that was artificially anchored to the plasma membrane by fusion with a membrane-binding domain, even in the absence of ROP11 (**Figure 2C**). These observations strongly suggest that MIDD1 mediates the restriction of active ROP11 by cortical microtubules (Oda and Fukuda, 2012a). The precise mechanism by which cortical microtubules restrict the ROP11-MIDD1 complex at the plasma membrane remains to be determined. Cortical microtubules are closely anchored to the plasma membrane by unknown MAPs, and it is thus likely that the active ROP11 protein complex, which includes MIDD1 and AtKinesin-13A, is restricted in its ability to diffuse through the space between the cortical microtubules and the plasma membrane. Consistent with this hypothesis is the elimination of an *Arabidopsis* formin, AtFH1, which has a large cytoplasmic domain, from the plasma membrane by cortical microtubules (Martiniere et al., 2011). Similarly, cortical actin microfilaments limit the diffusion of plasma membrane-anchored proteins in animal cells (Kusumi et al., 2005). Instead of actin filaments, plasma-membrane-associated cortical microtubules are used in plants as membrane fences to restrict localization of plasma membrane-anchored proteins.

## INTERPLAY BETWEEN MICROTUBULES AND PLASMA MEMBRANE DOMAINS MAY ESTABLISH PATTERNS

Evenly distributed active ROP11 domains are spontaneously generated by ROPGEF4, ROPGAP3, and ROP11, and recruit the MIDD1-AtKinesin-13A complex to the domains, which in turn depolymerizes cortical microtubules at the domains. The surrounding cortical microtubules limit the localization of ROP11-MIDD1 complex to the domains, probably by inhibiting the outward lateral diffusion of the ROP11-MIDD1 complex (**Figure 2D**). These two interactions cause a mutual inhibition between cortical microtubules and plasma membrane domains underlain by active ROP11, resulting in spatial restriction of cortical microtubules and the plasma membrane domains (**Figure 2E**). Variability in the balance between these two pathways may produce a range of secondary wall pit shapes. For example, if the restriction of plasma membrane domains by cortical microtubules is dominant, reticulate secondary walls with oval pits will be formed. Conversely, if the microtubule depolymerization activity of the plasma membrane domain becomes dominant, pitted secondary walls with round pits will be formed. Therefore, this interplay between the plasma membrane domains and cortical microtubules may be a key determining factor leading to different secondary wall patterns.

Similar interplay between plasma membrane domains and cortical microtubules is reported in leaf epidermal morphogenesis. In leaf epidermis, pavement cells grow to form an interdigitating structure where lobes and indentations develop side-by-side. ROP6 and its effector RIC1 recruit microtubule-severing katanin protein to facilitate the ordering of a parallel cortical microtubule array that restricts cell growth in indentations, while ROP2/4 and their effector RIC4 enhance cortical actin microfilaments to promote local growth of lobes (Fu et al., 2005, 2009; Lin et al., 2013). The cortical actin microfilaments inhibit endocytosis of an auxin efflux carrier PIN1, promoting accumulation of PIN1 protein at the lobe forming region, which in turn promotes accumulation of auxin, which then activates the ROP6-RIC1 pathway in its neighbor cell via auxin binding protein ABP1 (Xu et al., 2010; Nagawa et al., 2012). These two signaling pathways appear to spatially restrict one another: the ROP2/4-RIC4 pathway eliminates RIC1 from the lobe forming regions and inhibits parallel ordering of cortical microtubules, while the ROP6-RIC1 pathway inhibits the ROP2/4-RIC4 pathway, preventing co-existence of these two pathways in the same area. Although the precise mechanism by which these two pathways spatially restrict each other is still unclear, one possibility is that the parallel cortical microtubules in the indentation inhibit lateral diffusion of ROP2/4-RIC4 complex on the plasma membrane, in a similar manner to the restriction of the ROP11-MIDD1 complex by cortical microtubules. It is also possible that the ROP-MIDD-AtKinesin-13 pathway contributes to the elimination of cortical microtubules from lobes and that the dynamic interplay between cortical microtubules and plasma membrane domains of ROP GTPase might be widely utilized in plant cell morphogenesis.

## CONCLUDING REMARKS

Recent studies on xylem vessel differentiation and leaf epidermis morphogenesis revealed dynamic interplay between cortical microtubules and plasma membrane domains. ROP GTPases play critical roles in generating *de novo* plasma membrane domains and in regulating the function of the domains by recruiting different effector proteins and MAPs. In xylem cells, MIDD1 plays a central role in this interplay. Considering the diversity of the ROP GTPases and MIDD/RIP/ICR family members, the ROP-MIDD/RIP/ICR pathway may be involved in various cellular events by mediating interplay between plasma membrane domains and cortical microtubules. Recent proteomic approaches suggest that MIDD1/RIP/ICR members are also MAPs (Hamada et al., 2013). Further analysis of the pathways will provide novel insights regarding the molecular mechanisms underlying local control of plasma membrane domains coupled to microtubule dynamics in various plant cells.

## Conflict of Interest Statement

The authors declare that the research was conducted in the absence of any commercial or financial relationships that could be construed as a potential conflict of interest.
